# Ed accesses severity for intossication and substance abuse during the first pandemic wave of 2019 coronavirus pandemic (COVID-19). experience of a lombardy ed

**DOI:** 10.1192/j.eurpsy.2021.802

**Published:** 2021-08-13

**Authors:** G. Savioli, S. Pesenti, I. Ceresa, E. Oddone, M.A. Bressan

**Affiliations:** 1 Emergency Department, IRCCS Policlinico San Matteo, Pavia, Italy; 2 In Cammino Social Cooperative Of San Pellegrino Terme (bg)., La Bonne Semence social cooperative of Oltre il Colle (BG), Bergamo, Italy; 3 Department Of Public Health, University of Pavia, Pavia, Italy

**Keywords:** Emergency department, intossication and substance abuse, COVID-19 pandemic

## Abstract

**Introduction:**

The 2019 coronavirus epidemic (CoViD-19) in Italy originated in Lombardy, on February 21, 2020. The Fondazione IRCCS Policlinico San Matteo di Pavia has been involved in the management of the outbreak since its beginning

**Objectives:**

We evaluated all the population who went to the ED for intossication and substance abuse to assess the severity of cases evaluated as exit code and rate of hospitalization.

**Methods:**

We enrolled all patients accessing our ED for intossication and substance abuse form February 22 to May 1, 2020 and during the same period of the previous year.

**Results:**

We enrolled 142 patients. 41 in the CoViD period and 101 in 2019. The vital parameters, and sex were overlapping. patients during the pandemic were younger (38 vs 46) The priority codes for the medical examination were not different. CoViD pandemic patients have higher codes (yellow and red) for the medical examination (66% vs 59%); discharge severity codes (red) more frequently than in the reference period (2.4% vs 0.9%) and more frequently need hospitalization (26.8% vs 16.8%).

**Conclusions:**

The epidemic has led to a reduction of accesses for intossication and substance abuse. Patients had more frequent hospitalization needs and more severe exit codes. the data may be due to the fact that during the pandemic only the most serious patients access the E.D., but also to the fact that a pandemic has contributed to destabilizing this class of fragile patients.

